# Cysteinyl-leukotrienes in the regulation of β_2_-adrenoceptor function: an in vitro model of asthma

**DOI:** 10.1186/1465-9921-7-103

**Published:** 2006-07-28

**Authors:** G Enrico Rovati, Michele Baroffio, Simona Citro, Lorenzo Brichetto, Saula Ravasi, Manlio Milanese, Emanuele Crimi, Vito Brusasco

**Affiliations:** 1 Laboratory of Molecular Pharmacology, Section of Eicosanoid Pharmacology, Dept. of Pharmacological Sciences, University of Milan, Italy; 2 Respiratory Pathophysiology Unit, Dept. of Internal Medicine, University of Genoa, Italy

## Abstract

**Background:**

The response to β_2_-adrenoceptor agonists is reduced in asthmatic airways. This desensitization may be in part due to inflammatory mediators and may involve cysteinyl-leukotrienes (cysteinyl-LTs). Cysteinyl-LTs are pivotal inflammatory mediators that play important roles in the pathophysiology of asthma, allergic rhinitis, and other inflammatory conditions. We tested the hypothesis that leukotriene D_4 _(LTD_4_) and allergen challenge cause β_2_-adrenoceptor desensitization through the activation of protein kinase C (PKC).

**Methods:**

The isoproterenol-induced cAMP accumulation was evaluated in human airway smooth muscle cell cultures challenged with exogenous LTD_4 _or the PKC activator phorbol-12-myristate-13-acetate with or without pretreatments with the PKC inhibitor GF109203X or the CysLT_1_R antagonist montelukast. The relaxant response to salbutamol was studied in passively sensitized human bronchial rings challenged with allergen in physiological salt solution (PSS) alone, or in the presence of either montelukast or GF109203X.

**Results:**

In cell cultures, both LTD_4 _and phorbol-12-myristate-13-acetate caused significant reductions of maximal isoproterenol-induced cAMP accumulation, which were fully prevented by montelukast and GF109203X, respectively. More importantly, GF109203X also prevented the attenuating effect of LTD_4 _on isoproterenol-induced cAMP accumulation. In bronchial rings, both montelukast and GF109203X prevented the rightward displacement of the concentration-response curves to salbutamol induced by allergen challenge.

**Conclusion:**

LTD_4 _induces β_2_-adrenoceptor desensitization in human airway smooth muscle cells, which is mediated through the activation of PKC. Allergen exposure of sensitized human bronchi may also cause a β_2_-adrenoceptor desensitization through the involvement of the CysLT_1_R-PKC pathway.

## Background

Inhaled β_2_-adrenoceptor (β_2_-AR) agonists represent a first-line treatment of bronchial asthma. However, a reduced response to β_2_-AR agonists has been observed in asthmatic subjects and it has been suggested to play a role in airway hyperresponsiveness [1,2]. Although genetic factors may influence responses to β-agonists [3,4], it is believed that the reduced response of β_2_-AR may result from use of β-agonists leading to receptor desensitization [5,6]. Moreover, β_2_-AR desensitization can be induced in human airway smooth muscle cells (HASMC) by exposure to inflammatory mediators that are likely to be present in the asthmatic airways [7,8]. In allergic asthma, several products are released from either resident or circulating inflammatory cells or even from the HASMC themselves [9] upon exposure to allergen. Among these mediators, cysteinyl-leukotrienes (cysteinyl-LTs) are long known to play an important role in asthma [10,11]. Cysteinyl-LTs originate from the oxidative metabolism of arachidonic acid through 5-lipoxygenase in different inflammatory cells and are released upon exposure to sensitizing allergens [12,13]. Cysteinyl-LTs exert a variety of effects with relevance to the etiology of asthma [14], like smooth muscle contraction [15-17] and proliferation [18,19], eosinophil recruitment into the airways [20], increased microvascular permeability [21], enhanced mucus secretion and decreased mucus transport [12,22]. Furthermore, in passively sensitized human bronchi, the response to β_2_-AR agonists is reduced after allergen exposure, and this can be prevented by either a cell membrane stabilizer or a leukotriene receptor antagonist, suggesting a role for cysteinyl-LTs released by resident inflammatory cells regulating β_2_-AR function [23]. Consistent with this hypothesis is the clinical observation that concurrent administration of salbutamol and the CysLT_1_receptor (CysLT_1_R) antagonist montelukast affords greater protection against exercise- and hyperventilation-induced asthma than salbutamol alone [24].

The intracellular mechanisms through which cysteinyl-LTs may cause β_2_-AR desensitization in asthmatic airways have not been fully investigated. In the present study, we tested the hypothesis that cysteinyl-LTs may cause β_2_-AR desensitization through the activation of protein kinase C (PKC). For this purpose, the isoproterenol-induced cAMP production was first studied in HASMC pre-incubated with exogenous LTD_4 _or the PKC activator phorbol-12-myristate-13-acetate (PMA). Then, the effects of montelukast and the specific PKC inhibitor GF109203X were compared in LTD_4_-challenged HASMC. Possible effects of LTD_4 _on protein kinase A (PKA) or adenylyl-cyclase were assessed by treatments with the PKA inhibitor H89 or forskolin. The hypothesis that the LTD_4_-PKC pathway may also be involved for allergen-induced β_2_-AR desensitization was tested by assessing the effects of montelukast and GF109203X in passively sensitized human bronchial rings challenged with allergen.

## Methods

### Materials

Smooth muscle cells from human bronchi were purchased from Invitrogen-Cambrex (Walkersville, MD). Cell culture supplies, forskolin, PMA, isobutylmethylxanthine (IBMX) and isoproterenol were purchased from Sigma Chemical Co (St. Louis, MO); LTD_4 _and cAMP EIA kit from Cayman Chemical Co. (Ann Arbor, MI); montelukast was a gift from Merck & Co. (West Point, PA). GF109203X and H89 were from Calbiochem (La Jolla, CA). DC™Protein assay from Bio-Rad Laboratories (Richmond, CA). Bronchial rings for functional studies were obtained from 6 non-asthmatic patients undergoing thoracotomy for lung cancer.

### HASMC studies

Monolayers of HASMC from human bronchi were grown in Minimum Essential Medium supplemented with 10% FBS, 100-U/ml penicillin, and 100-μg/ml streptomycin, as previously described in detail [25]. Cells were used between 3^rd ^and 8^th ^passage at a 1:3 ratio in 75-cm^2 ^culture flasks. At least two different cell line have been used.

Accumulation of cAMP was measured in cells grown to confluence in 12-well plates and serum-starved for 24 h. Cells were incubated at 37°C for 10 min in 1-ml PBS containing 3 × l0^-4^M ascorbic acid and 10^-3^M isobutylmethylxanthine. Reactions were stopped by placing the plates on ice, cells were then washed once with cold PBS and 150 μl of l0^-1^M HC1 were added to each well. After 20-min incubation, cells were scraped and centrifuged 12000 × g for 10 min. Supernatant solutions were first assayed for protein concentration and then for cAMP content using a cAMP EIA-kit following manufacturer's instructions. cAMP concentrations of unknown samples were determined by computer-assisted interpolation from a standard curve.

Concentration-response curves of cAMP accumulation in response to isoproterenol (10^-9^M to 10^-4^M) were obtained in HASMC at control (vehicle treated) or after exposure to LTD_4 _(10^-6^M for 30 min), with or without 30-min pre-incubation with 10^-6^M GF109203X. The increase of cAMP above baseline in response to 10^-5^M isoproterenol was studied in HASMC at control and after 30-min exposure to 10^-6^M LTD_4 _or 5 × l0^-7^M PMA, with or without 10^-6^M montelukast, GF109203X, or H89. The effect of 10^-4^M forskolin was studied by 10-min incubation after 30-min exposure to either vehicle or LTD_4_.

### Bronchial tissue studies

24 bronchial rings from surgical specimens were passively sensitized against dust mites by an overnight incubation (18 h) at room temperature with serum pooled from three atopic subjects diluted 1:9 in aerated (95% O_2_, 5 % CO_2_) PSS of the following composition (mM): NaCl 110.5, KC1 3.4, CaCl_2 _2.4, MgSO_4 _0.8, KH_2_PO_4 _1.2, NaHCO_3 _25.7, and dextrose 5.6, as previously described in details [26]. The serum specific concentrations of specific IgE for Dermatophagoides Pteronyssinus and D. Farinae were larger than 13.2 Phadebast RAST units/ml (Pharmacia, Uppsala, Sweden) and the total serum concentration was 180 ± 33 international units/ml. Nineteen sensitized rings were incubated with montelukast (10^-7^M, n = 5 and 10^-6^M, n = 5), or GF109203X (10^-7^M, n = 2 and 10^-6^M, n = 1), or PSS (n = 6) for 30 min and then challenged by a 60-min incubation with 200 AU/ml of Dermatophagoides mix at 37°C. Challenged rings incubated with PSS alone served as control (n = 6). Rings were then suspended in water-jacketed 25-ml tissue baths containing aerated PSS at 37°C using two stirrups connected to a fixed hook at the bottom of the tissue bath and to a force transducer via a silk string, respectively. Rings were gradually stretched until a steady reference length of 1 gr was achieved. PSS was changed every 20 min. All rings were contracted with 10^-6^M carbachol and, after a steady contraction was achieved, relaxed with salbutamol added cumulatively from 10^-9^M to 10^-4^M with half-Log increments. Each concentration-response curve was fitted by sigmoid least-square interpolation between extreme values constrained at 100% (maximal carbachol-induced force) and 0 (minimal force at 10^-4^M salbutamol).

### Statistical analysis and experimental design

All curves shown were analyzed by Prism-4 software using the four parameters logistic equation and parameters compared using the extra sum of square principle [27]. Parameter errors are expressed as percentage coefficient of variation (%CV) and calculated by simultaneous analysis of at least two different and independent experiments performed in duplicate or triplicate (for HASMC). One-way independent or two-way repeated-measure analysis of variance (ANOVA) were used whenever appropriate with Dunnett or Bonferroni post-hoc tests for multiple comparisons. P values < 0.05 were considered statistically significant. Data are expressed as means ± S.E.M.

## Results

### Isoproterenol-induced cAMP accumulation in HASMC culture

Increasing concentrations of isoproterenol caused a concentration-dependent accumulation of cAMP in all experiments.

**Figure 1:**
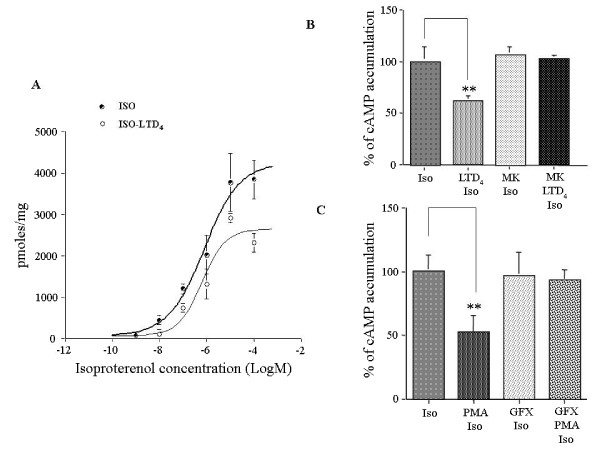
**Effect of exogenous LTD_4 _or PMA challenge on isoproterenol-induced cAMP accumulation in HASMC**. *A-B*. Effects of leukotriene D_4 _(LTD_4_, 10^-6^M) challenge and pretreatment with the CysLT_1_R antagonist montelukast (MK, 10^-6^M) on cAMP accumulation induced by multiple (*A*) and single (*B*, 10^-5^M) isoproterenol concentrations in HASMC. *C*. Effect of phorbol-12-myristate-13-acetate (PMA, 5 × l0^-7^M) challenge and pretreatment with the PKC inhibitor GF109203X (10^-6^M) on cAMP accumulation induced by 10^-5^M isoproterenol in HASMC. The results are presented as mean ± S.E.M. of at least three experiments performed in triplicate. **P < 0.01 (one-way ANOVA).

After challenge with LTD_4 _(Fig. 1A) the maximum cAMP accumulation was significantly (P < 0.05) reduced (33%) from 4109 pmol/mg prot (CV 10%) to 2760 pmoles/mg prot (CV 13%), whereas EC_50 _was substantially unaffected (from 0.68 μM, CV 59% to 0.69 μM, CV 82%). In montelukast-treated and LTD_4_-challenged HASMC (Fig. 1B), isoproterenol-induced cAMP accumulation was not significantly different from unchallenged HASMC and significantly greater than in untreated LTD_4_-challenged HASMC (P < 0.01).

After challenge with PMA (Fig. 1C), the maximum isoproterenol-induced cAMP accumulation was significantly (P < 0.01) reduced to 52% ± 12 SEM of the maximal stimulation, suggesting that PKC plays a pivotal role in the regulation of β_2_-AR in HASMC. In GF109203X-treated and PMA-challenged HASMC, isoproterenol-induced cAMP accumulation was not significantly different from unchallenged HASMC and significantly greater than in untreated PMA-challenged HASMC (P < 0.01).

**Figure 2:**
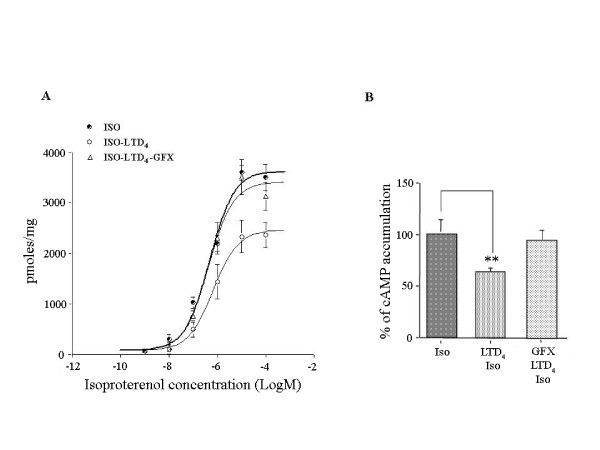
**Effect of exogenous LTD_4 _challenge and pretreatment with GFX109203X on isoproterenol-induced cAMP accumulation in HASMC**. Effects of LTD_4 _(10^-6^M) challenge and pretreatment with GF109203X (10^-6^M) on cAMP accumulation induced by multiple (*A*) and single (*B*, 10^-5^M) isoproterenol concentrations in HASMC. The results are presented as mean ± S.E.M. of at least three experiments performed in triplicate. **P < 0.01 (one-way ANOVA).

More importantly, in GF109203X-treated and LTD_4_-challenged HASMC (Fig. 2) the maximal isoproterenol-induced cAMP accumulation was 3417 pmoles/mg prot (CV 5%), significantly (P < 0.01) greater than 2464 pmoles/mg prot (CV 7%) in untreated LTD_4_-challenged HASMC and insignificantly different from 3632 pmol/mg prot (CV 5%) in unchallenged HASMC, confirming a critical role for PKC in the LTD_4_-induced β_2_-AR desensitization.

**Figure 3:**
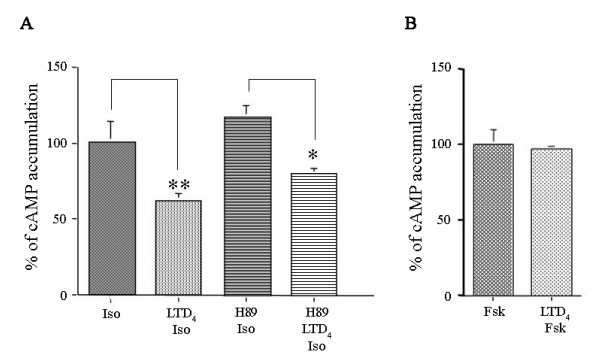
**Effect of exogenous LTD_4 _challenge on isoproterenol- or forskolin-induced cAMP accumulation in HASMC**. *A*. Effects of LTD_4 _(10^-6^M) challenge and pretreatment with the PKA inhibitor H89 (10^-6^M) on cAMP accumulation induced by single (10^-5^M) isoproterenol concentration in HASMC. *B*. Effects of LTD_4 _(10^-6^M) challenge on cAMP accumulation induced by single (10^-4^M) forskolin concentration in HASMC. The results are presented as mean ± S.E.M. of at least two experiments performed in triplicate. **P < 0.01, *P < 0.05 (oneway ANOVA).

Pre-treatment with H89 did not alter the effect of LTD_4 _challenge on isoproterenol-induced maximal cAMP accumulation (Fig. 3A), suggesting that LTD_4_-induced β_2_-AR desensitization does not involve PKA activation. Moreover, LTD_4 _challenge did not affect the forskolin-induced maximal cAMP accumulation (Fig. 3B), suggesting that the adenylyl cyclase was not directly affected by LTD_4_.

### Relaxant responses to salbutamol in human bronchial rings

The mean weight of the 24 bronchial rings was 91 ± 5 mg. The mean resting force and the mean normalized-response to carbachol were 0.83 ± 0.05 g and 14 ± 2 gr/gr of tissue, without significant differences between sensitized, challenged, and treated rings Table 1.

**Table 1:** Physical and mechanical characteristics of the human bronchial rings used for different experiments.

condition	n	muscle weight, g	resting force	CCh response, g/g of tissue
sensitized	5	72 ± 4	0.72 ± 0.04	12 ± 3
challenged	6	77 ± 4	0.73 ± 0.11	12 ± 3
MLK 10^-7^M	5	102 ± 14	0.91 ± 0.05	19 ± 5
MLK 10^-6^M	5	109 ± 9	0.84 ± 0.15	14 ± 3
GFX 10^-7^M	2	116; 99	0.96; 1.23	31; 10
GFX 10^-6^M	1	96	1.05	8

Data are mean ± s.e.m. or individual values.

**Figure 4:**
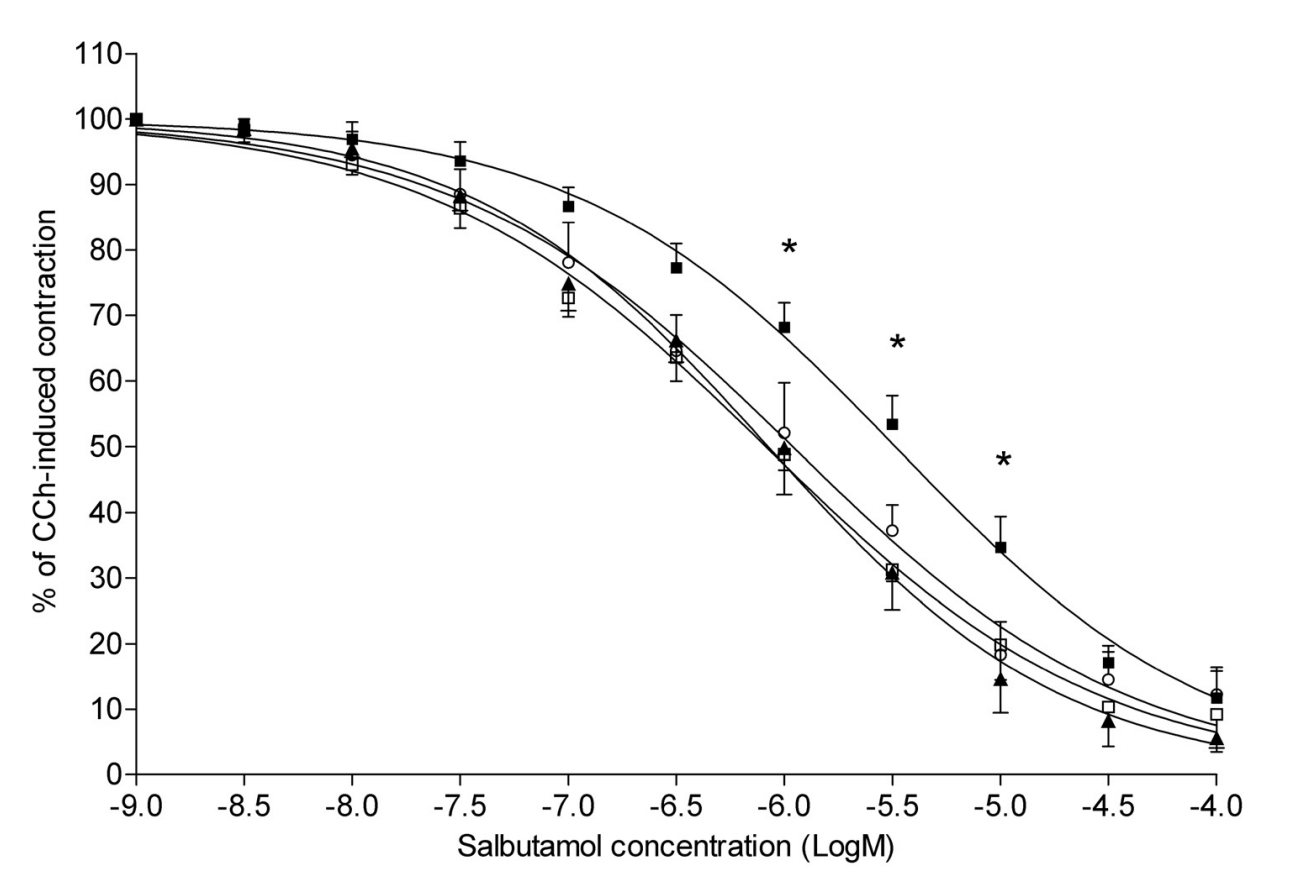
**Effect of the pretreatment with montelukast on salbutamol-induced relaxation in challenged human bronchial rings**. Relaxant responses to salbutamol in carbachol-contracted human bronchial rings. Values of 100 and 0 on y-axis represent maximal force in response to 10^-6^M carbachol and minimal force at 10^-4^M salbutamol, respectively. ▲, sensitized control rings (n = 5); ■, challenged-untreated rings (n = 6); ○, montelukast 10^-7^M-treated rings (n = 5); □, montelukast 10^-6^M-treated rings (n = 5). The results are presented as mean ± S.E.M. *P < 0.05 (two-way repeated-measure ANOVA followed by Bonferroni post-hoc test) ■ *vs*. ▲, ○, and □.

Salbutamol relaxed bronchial rings significantly (P < 0.01) in a concentration-dependent manner (Fig. 4). The salbutamol concentration-response curve of challenged rings was significantly (P < 0.01) shifted to the right of the dose response curve of sensitized unchallenged rings, with significant differences (P < 0.01) at salbutamol concentrations from 10^-6^M to 10^-5^M. Pre-treatment with either 10^-6^M or 10^-7^M montelukast displaced significantly (P < 0.01) to the left of the concentration-response curves of challenged rings, with significant differences (P < 0.05) at salbutamol concentrations from 10^-6^M to 10^-5^M.

**Figure 5:**
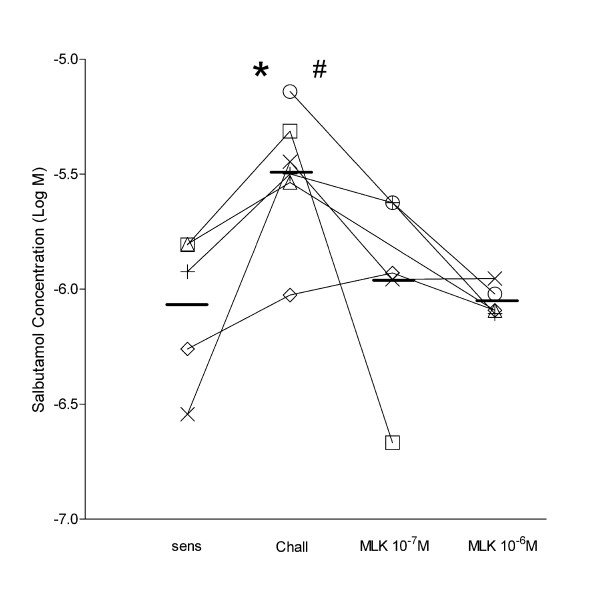
**Salbutamol concentrations inhibiting 50% of active force in carbachol-contracted human bronchial rings**. Effects of montelukast on salbutamol concentrations inhibiting 50% of carbachol-induced contraction (IC_50_). * P < 0.05 challenged *vs*. sensitized and montelukast 10^-6^M, # P = 0.07 challenged *vs*. montelukast 10^-7^M (one-way ANOVA followed by Bonferroni post-hoc test). Each symbol represent rings from the same subject.

The mean values for IC_50 _of challenged rings was -5.49 ± 0.12 Log M significantly (P < 0.05) higher than -6.07 ± 0.15 Log M of sensitized untreated rings (Fig. 5). The IC_50 _values of challenged rings treated with 10^-6^M and 10^-7^M montelukast were -6.05 ± 0.03 and 5.96 ± 0.19, respectively, which were not significantly different from those of sensitized untreated rings. The IC_50 _values of challenged rings treated with montelukast were lower than those of challenged rings (P < 0.05 for 10^-6^M and P = 0.07 for 10^-7^M).

**Figure 6:**
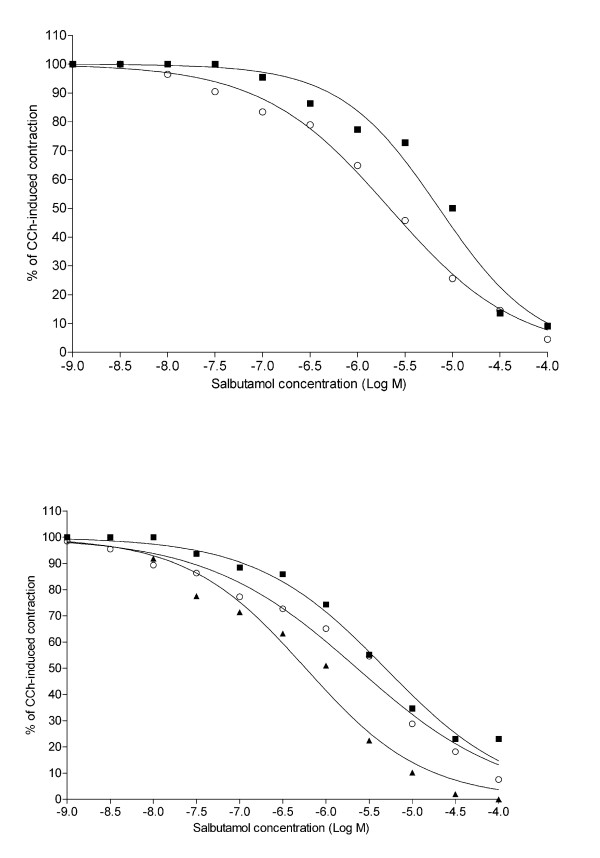
**Effect of the pretreatment with the PKC inhibitor GF109203X on salbutamol-induced relaxation in challenged human bronchial rings**. Relaxant responses to salbutamol in five carbachol-contracted human bronchial rings. Values of 100 and 0 on y-axis represent maximal force in response to 10^-6^M carbachol and minimal force at 10^-4^M salbutamol, respectively. ■, challenged-untreated rings; ○, rings pre-treated with 10^-7^M GF109203X; ▲, ring pre-treated with 10^-6^M GF109203X.

In challenged rings treated with either 10^-7^M or 10^-6^M GF109203X, the concentration-response curves to salbutamol were significantly (P < 0.01) shifted to the left of the concentration-response curve of challenged rings (Fig. 6).

## Discussion

The major findings of the present study can be summarized as follows: 1) In HASMC, exogenous LTD_4 _caused a reduction of isoproterenol-induced cAMP accumulation similar to that caused by direct activation of PKC, 2) this effect of LTD_4 _was prevented not only by the CysLT_1_R antagonist montelukast, but also by direct inhibition of PKC, and 3) both montelukast and direct PKC inhibition prevented the reduction of response to salbutamol caused by allergen challenge of passively sensitized human bronchi.

### Comments on methodology

**Figure 7:**
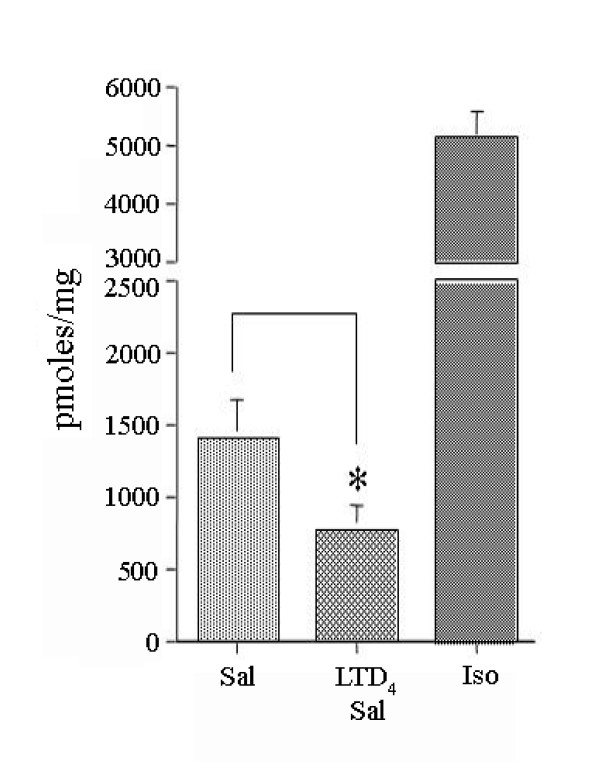
**Effect of exogenous LTD_4 _challenge on salbutamol-induced cAMP accumulation in HASMC**. Effect of LTD_4 _(10^-6^M) challenge on cAMP accumulation induced by 10^-4^M salbutamol in HASMC. Note the weaker effect of salbutamol compare to isoproterenol (10^-5 ^M) and the similarity with the effects of LTD_4 _in Fig.s 1-3. The results are presented as mean ± s.e.m. of two experiments performed in triplicate. *P < 0.01 (one-way ANOVA).

We first constructed concentration response curves of isoproterenol-induced cAMP accumulation in HASMC utilizing a non-cumulative protocol. A maximum effect was clearly observed at isoproterenol concentration of 10^-5^M, and this was therefore used for subsequent single-concentration experiments. Isoproterenol was used in cAMP accumulation experiments because, as a full β-AR agonist, is more suited for the desensitization studies. The β_2_-AR selective partial agonist salbutamol was used for bronchial rings studies because it is the reference drug generally used for clinical studies. However, in two separate experiments we found that the effect of salbutamol on cAMP accumulation was much weaker than that of isoproterenol, while the relative reduction caused by LTD_4 _challenge was similar to that observed using isoproterenol, being even slightly more pronounced (Fig. 7). Therefore, we are confident that the results of our HASMC and bronchial rings studies are comparable.

Furthermore, the fact that after LTD_4 _challenge in HASMC only the maximal cAMP accumulation was reduced, whereas only the IC_50 _of salbutamol-induced relaxation was reduced might be explained by the fact that the relaxing effect of a β2 agonist is a far more downstream response than a second messenger (i.e. cAMP) production, and certainly involve the activation of other components downstream of the receptor, while the β2-AR may perform functions other than adenylyl cyclase activation [28], yet equally involved in bronchial relaxation.

As in our previous studies [23,26,29-31], human bronchial rings were passively sensitized by using a pool of sera containing high levels of specific IgEs but low levels of total IgEs. With this method of passive sensitization and allergen challenge, followed by repeated washouts, the force generation capacity of airway smooth muscle was not altered [23], which makes us confident that the reference force of 1 g and the level of pre-contraction induced by carbachol 10^-6^M were similar in all experimental conditions. Furthermore, the relaxant responses to either theophylline [26] or forskolin [30] remained unaltered in previous studies using the same methodology. Therefore, the use of sensitized unchallenged rings as a control seems justified and any difference in response to salbutamol can be attributed to changes in the β_2_-AR pathway.

For relaxation studies, bronchial rings were pre-contracted with the non-selective muscarinic agonist carbachol, thus activating both M_3 _and M_2 _receptors on smooth muscle cell membrane. M_2 _receptors are coupled to G_i_-protein, which inhibits adenylyl cyclase. Thus, had sensitization or allergen challenge changed G_i_-protein expression or activity, the response to a β_2_-agonist would have been affected. In this model, however, both expression and activity of G_i_-protein were similar in sensitized and challenged rings [29].

In bronchial tissue studies, the effects of allergen challenge were presumably due to mediator release from resident inflammatory cells [23]. Thus, it cannot be excluded that the protective effects of GFX and montelukast against β_2_-AR dysfunction were in part due to inhibition of mediator release. However, the observation that GFX and montelukast also protected against β_2_-AR dysfunction in HASMC does suggest that airway smooth muscle PKC was directly involved

### Comments on results

The response to β_2_-AR has been found to be reduced in airways from subjects with fatal asthma [32]. A reduced β_2_-AR responsiveness in asthma may be the result of activation of the β_2_-AR by specific agonists (homologous desensitization) or activation of other receptors by the inflammatory mediators, which are present in the asthmatic airways (heterologous desensitization) [33]. β_2_-AR desensitization induced by agents that increase cAMP levels, such as bradykinin [34] and some cytokines [35] acting through the elevation of prostaglandin E_2 _[36], is probably regulated by PKA [6,33]. On the contrary, muscarinic agonists [37], phorbol esters, and other inflammatory mediators may attenuate responses to β-agonists through the activation of PKC [38], as also recently suggested in bovine tracheal smooth muscle preparations [39,40]. However, it appears that these mechanisms of desensitization are cell-type specific [41] and may depend on kinase expression levels [42].

Among the inflammatory mediators involved in asthma, cysteinyl-LTs seem to play a key role in the bronchoconstrictor response to allergen [15-17] through activation of CysLT_1_R. Though preferentially coupled to G_q/11_-protein, constitutively expressed CysLT_1 _also activates pertussis toxin (PTX)-sensitive and -insensitive G-proteins [43,44]. In HASMC, we have previously found that CysLT_1 _stimulation activates PKC [25] and mitogen-activated protein kinases ERK1/2 through mechanisms that involve a PTX-sensitive G-protein [19]. Thus, it is possible that cysteinyl-LTs may contribute to β_2_-AR desensitization not only by a PKC-dependent mechanism, but also by modulating the adenylyl cyclase-PKA pathway.

The results of the present study show that the cAMP accumulation in response to isoproterenol is reduced in HASMC treated with exogenous LTD_4 _or the PKC activator PMA and the relaxant response to salbutamol is reduced in human bronchi challenged with the sensitizing allergen. The effects of LTD_4 _in HASMC and allergen challenge in bronchial rings were prevented by the CysLT_1_R antagonist montelukast and the PKC specific inhibitor GF109203X. Altogether, these findings strongly suggest that in the models used in the present study β_2_-AR desensitization was the result of PKC activation by LTD_4_.

In HASMC, exogenous LTD_4 _did not alter the cAMP accumulation induced by forskolin, thus excluding that the reduced response of β_2_-AR to isoproterenol was due to adenylyl cyclase dysfunction. The PKA inhibitor H89 also failed to prevent the LTD_4_-induced β_2_-AR desensitization in HASMC, thus ruling out the possibility of the involvement of this protein kinase. Indeed, H89 tended to enhance the response to isoproterenol both in LTD_4_-challenged and -unchallenged HASMC, suggesting the presence of the well known G_S_/G_i _switch phenomenon of β_2_-AR coupling due to PKA phosphorylation [45], which was not enhanced by LTD_4_. This finding suggests that the β_2_-AR function is independently modulated by PKA and PKC mechanisms and it is consistent with the observations by Penn et al. [6] who showed that inhibition of PKC did not alter β_2_-AR desensitization induced by PKA activation.

In human bronchi, allergen challenge may cause β_2_-AR desensitization through different mechanisms involving inflammatory mediators other than LTs, thus possibly involving PKA. However, in previous studies we found that the reduction of relaxant response to salbutamol in allergen-challenged rings was not prevented by inhibition of prostaglandins [23], IL-lβ, or TNFα [30], which are known to cause β_2_-AR dysfunction/desensitization through the activation of PKA [6,33,35,36].

## Conclusion

In conclusion, taken together these data suggest that cysteinyl-LTs cause desensitization of β_2_-AR in both HASMC and isolated human bronchi through an acute mechanism involving PKC but not PKA, and that this desensitization might be prevented by the CysLT_1_R antagonist montelukast. If cysteinyl-LTs released from resident or circulating inflammatory cells or even from the smooth muscle cell itself are the major responsible for β_2_-AR desensitization in asthma, then the concurrent administration of CysLT_1_R antagonists may represent a useful tool to improve the response to β_2_-AR agonists in this disease. Clinical trials are necessary to assess the efficacy of the association between CysLT_1_R antagonists and β_2_-AR agonists in bronchial asthma.

## Competing interests

GER received a research grant in 2005 from Merck, Sharpe & Dohme for in vitro studies on montelukast.

MB declare no competing interests.

SC declare no competing interests.

LB declare no competing interests.

SR declare no competing interests.

MM declare no competing interests

EC declare no competing interests

VB received a research grant in 2004 from Merck, Sharpe & Dohme for in vitro studies on montelukast.

## Authors' contributions

GER conceived and designed the study, coordination and manuscript preparation. MB was involved in isolated human bronchial ring experiments and helped in manuscript preparation.

SC participated in the design of the experiments, was involved in HASMC culture, performed in vitro cAMP studies and helped in the manuscript preparation.

LB participated in the design of the experiments and was involved in isolated human bronchial ring experiments.

SR participated to the in vitro studies.

MM was involved in isolated human bronchial ring experiments.

EC participated in the design and coordination of the experiments.

VB conceived and designed the study and participated to the manuscript preparation.

## References

[B1] Emala C, Black C, Curry C, Levine MA, Hirshman CA (1993). Impaired beta-adrenergic receptor activation of adenylyl cyclase in airway smooth muscle in the basenji-greyhound dog model of airway hyperresponsiveness. Am J Respir Cell Mol Biol.

[B2] Nielson CP, Crowley JJ, Vestal RE, Connolly MJ (1992). Impaired beta-adrenoceptor function, increased leukocyte respiratory burst, and bronchial hyperresponsiveness. J Allergy Clin Immunol.

[B3] Israel E, Drazen JM, Liggett SB, Boushey HA, Cherniack RM, Chinchilli VM, Cooper DM, Fahy JV, Fish JE, Ford JG (2001). Effect of polymorphism of the beta(2)-adrenergic receptor on response to regular use of albuterol in asthma. Int Arch Allergy Immunol.

[B4] Rathz DA, Gregory KN, Fang Y, Brown KM, Liggett SB (2003). Hierarchy of polymorphic variation and desensitization permutations relative to beta 1- and beta 2-adrenergic receptor signaling. J Biol Chem.

[B5] Hausdorff WP, Caron MG, Lefkowitz RJ (1990). Turning off the signal: desensitization of beta-adrenergic receptor function. FASEB J.

[B6] Perm RB, Panettieri RA, Benovic JL (1998). Mechanisms of acute desensitization of the beta2AR-adenylyl cyclase pathway in human airway smooth muscle. Am J Respir Cell Mol Biol.

[B7] Benovic JL (2002). Novel beta2-adrenergic receptor signaling pathways. J Allergy Clin Immunol.

[B8] Shore SA, Moore PE (2002). Effects of cytokines on contractile and dilator responses of airway smooth muscle. Clin Exp Pharmacol Physiol.

[B9] Hirst SJ (2003). Regulation of airway smooth muscle cell immunomodulatory function: role in asthma. Respir Physiol Neurobiol.

[B10] Drazen JM, Austen KF (1987). Leukotrienes and airway responses. Am Rev Respir Dis.

[B11] Nicosia S, Capra V, Rovati GE (2001). Leukotrienes as mediators of asthma. Pulm Pharmacol Ther.

[B12] Samuelsson B (1983). Leukotrienes: mediators of immediate hypersensitivity reactions and inflammation. Science.

[B13] Dahlen SE, Hansson G, Hedqvist P, Björck T, Granström E, Dahlen B (1983). Allergen challenge of lung tissue from asthmatics elicits bronchial contraction that correlates with the release of leukotrienes C_4_, D_4 _and E_4_. Proc Natl Acad Sci USA.

[B14] Holgate ST, Peters-Golden M, Panettieri RA, Henderson WR (2003). Roles of cysteinyl leukotrienes in airway inflammation, smooth muscle function, and remodeling. J Allergy Clin Immunol.

[B15] Dahlen SE, Hedqvist P, Hammarstrom S, Samuelsson B (1980). Leukotrienes are potent costrictors of human bronchi. Nature.

[B16] Weiss JW, Drazen JM, Coles N, McFadden ER, Weller PF, Corey EJ, Lewis RA, Austen KF (1982). Bronchoconstrictor effects of leukotriene C in humans. Science.

[B17] Adelroth E, Morris MM, Hargreave FE, O'Byrne PM (1986). Airway responsiveness to leukotrienes C4 and D4 and to methacholine in patients with asthma and normal controls. N Engl J Med.

[B18] Panettieri RA, Tan EM, Ciocca V, Luttmann MA, Leonard TB, Hay DW (1998). Effects of LTD_4 _on human airway smooth muscle cell proliferation, matrix expression, and contraction In vitro: differential sensitivity to cysteinyl leukotriene receptor antagonists. Am J Respir Cell Mol Biol.

[B19] Ravasi S, Citro S, Viviani B, Capra V, Rovati GE (2006). CysLTl receptor-induced human airway smooth muscle cells proliferation requires ROS generation, EGF receptor transactivation and ERK1/2 phosphorylation. Respir Res.

[B20] Salvi SS, Krishna MT, Sampson AP, Holgate ST (2001). The anti-inflammatory effects of leukotriene-modifying drugs and their use in asthma. Chest.

[B21] Dahlen SE, Biörk J, Hedqvist P, Arfors KE, Hammarstrom S, Lindgren JA, Samuelsson B (1981). Leukotrienes promote plasma leakage and leukocyte adhesion in postcapillary venules: in vivo effects with relevance to the acute inflammatory response. Proc Natl Acad Sci USA.

[B22] Arm JP (2004). Leukotriene generation and clinical implications. Allergy Asthma Proc.

[B23] Song P, Crimi E, Milanese M, Duan J, Rehder K, Brusasco V (1998). Anti-inflammatory agents and allergen-induced beta2-receptor dysfunction in isolated human bronchi. Am J Respir Crit Care Med.

[B24] Coreno A, Skowronski M, West E, El-Ekiaby A, McFadden ER (2005). Bronchoprotective effects of single doses of salmeterol combined with montelukast in thermally induced bronchospasm. Chest.

[B25] Accomazzo MR, Rovati GE, Vigano T, Hernandez A, Bonazzi A, Bolla M, Fumagalli F, Viappiani S, Galbiati E, Ravasi S (2001). Leukotriene D4-induced activation of smooth-muscle cells from human bronchi is partly Ca2+-independent. Am J Respir Crit Care Med.

[B26] Song P, Milanese M, Crimi E, Rehder K, Brusasco V (1997). Allergen challenge of passively sensitized human bronchi alters M2 and beta2 receptor function. Am J Respir Crit Care Med.

[B27] Draper NR, Smith H (1966). Applied regression analysis.

[B28] Lefkowitz RJ (1998). G protein-coupled receptors. III. New roles for receptor kinases and beta-arrestins in receptor signaling and desensitization. J Biol Chem.

[B29] Song P, Milanese M, Crimi E, Bruzzone S, Zocchi E, Rehder K, Brusasco V (2000). G(s) protein dysfunction in allergen-challenged human isolated passively sensitized bronchi. Am J Physiol Lung Cell Mol Physiol.

[B30] Brichetto L, Milanese M, Song P, Patrone M, Crimi E, Rehder K, Brusasco V (2003). Beclomethasone rapidly ablates allergen-induced beta 2-adrenoceptor pathway dysfunction in human isolated bronchi. Am J Physiol Lung Cell Mol Physiol.

[B31] Milanese M, Riccio AM, Gamalero C, De Giovanni B, Brichetto L, Baroffio M, Crimi E, Brusasco V, Canonica GW (2005). A model of allergen-driven human airway contraction: beta2 pathway dysfunction without cytokine involvement. Ann Allergy Asthma Immunol.

[B32] Bai TR (1990). Abnormalities in airway smooth muscle in fatal asthma. Am Rev Respir Dis.

[B33] Bunemann M, Lee KB, Pals-Rylaarsdam R, Roseberry AG, Hosey MM (1999). Desensitization of G-protein-coupled receptors in the cardiovascular system. Ann Rev Physiol.

[B34] Pang L, Holland E, Knox AJ (1998). Impaired cAMP production in human airway smooth muscle cells by bradykinin: role of cyclooxygenase products. Am J Physiol.

[B35] Pascual RM, Billington CK, Hall IP, Panettieri RA, Fish JE, Peters SP, Perm RB (2001). Mechanisms of cytokine effects on G protein-coupled receptor-mediated signaling in airway smooth muscle. Am J Physiol Lung Cell Mol Physiol.

[B36] Laporte JD, Moore PE, Panettieri RA, Moeller W, Heyder J, Shore SA (1998). Prostanoids mediate IL-lbeta-induced beta-adrenergic hyporesponsiveness in human airway smooth muscle cells. Am J Physiol.

[B37] Grandordy BM, Mak JC, Barnes PJ (1994). Modulation of airway smooth muscle beta-adrenoceptor function by a muscarinic agonist. Life Sci.

[B38] Pitcher J, Lohse MJ, Codina J, Caron MG, Lefkowitz RJ (1992). Desensitization of the isolated beta 2-adrenergic receptor by beta-adrenergic receptor kinase, cAMP-dependent protein kinase, and protein kinase C occurs via distinct molecular mechanisms. Biochemistry.

[B39] Boterman M, Elzinga CR, Wagemakers D, Eppens PB, Zaagsma J, Meurs H (2005). Potentiation of beta-adrenoceptor function in bovine tracheal smooth muscle by inhibition of protein kinase C. Eur J Pharmacol.

[B40] Boterman M, Smits SR, Meurs H, Zaagsma J (2006). Protein kinase C potentiates homologous desensitization of the beta2-adrenoceptor in bovine tracheal smooth muscle. Eur J Pharmacol.

[B41] Shin M, Malbon CC (1994). Oligodeoxynucleotides antisense to mRNA encoding protein kinase A, protein kinase C, and beta-adrenergic receptor kinase reveal distinctive cell-type-specific roles in agonist-induced desensitization. Proc Natl Acad Sci USA.

[B42] McGraw DW, Liggett SB (1997). Heterogeneity in beta-adrenergic receptor kinase expression in the lung accounts for cell-specific desensitization of the beta2-adrenergic receptor. J Biol Chem.

[B43] Capra V, Accomazzo MR, Ravasi S, Parenti M, Macchia M, Nicosia S, Rovati GE (2003). Involvement of prenylated proteins in calcium signaling induced by LTD4 in differentiated U937 cells. Prostaglandins Other Lipid Mediat.

[B44] Capra V, Ravasi S, Accomazzo MR, Parenti M, Rovati GE (2004). CysLTl signal transduction in differentiated U937 cells involves the activation of the small GTP-binding protein Ras. Biochem Pharmacol.

[B45] Daaka Y, Luttrell LM, Lefkowitz RJ (1997). Switching of the coupling of the beta2-adrenergic receptor to different G proteins by protein kinase A. Nature.

